# Effects of creatine supplementation on memory in healthy individuals: a systematic review and meta-analysis of randomized controlled trials

**DOI:** 10.1093/nutrit/nuac064

**Published:** 2022-08-19

**Authors:** Konstantinos Prokopidis, Panagiotis Giannos, Konstantinos K Triantafyllidis, Konstantinos S Kechagias, Scott C Forbes, Darren G Candow

**Affiliations:** is with the Department of Musculoskeletal Biology, Institute of Life Course and Medical Sciences, University of Liverpool, Liverpool, United Kingdom; are with the Society of Meta-Research and Biomedical Innovation, London, United Kingdom; are with the Society of Meta-Research and Biomedical Innovation, London, United Kingdom; is with the Department of Life Sciences, Faculty of Natural Sciences, Imperial College London, London, United Kingdom; are with the Society of Meta-Research and Biomedical Innovation, London, United Kingdom; is with the Department of Nutrition & Dietetics, Musgrove Park Hospital, Taunton & Somerset NHS Foundation Trust, Taunton, United Kingdom; are with the Society of Meta-Research and Biomedical Innovation, London, United Kingdom; is with the Department of Metabolism, Digestion and Reproduction, Faculty of Medicine, Imperial College London, London, United Kingdom; is with the Department of Obstetrics & Gynaecology, Chelsea and Westminster Hospital NHS Foundation Trust, London, United Kingdom; is with the Department of Physical Education Studies, Faculty of Education, Brandon University, Brandon, Manitoba, Canada; is with the Faculty of Kinesiology and Health Studies, University of Regina, Regina, Saskatchewan, Canada

**Keywords:** ageing, cognition, creatine monohydrate, memory, nutrition

## Abstract

**Context:**

From an energy perspective, the brain is very metabolically demanding. It is well documented that creatine plays a key role in brain bioenergetics. There is some evidence that creatine supplementation can augment brain creatine stores, which could increase memory.

**Objective:**

A systematic review and meta-analysis of randomized controlled trials (RCTs) was conducted to determine the effects of creatine supplementation on memory performance in healthy humans.

**Data Sources:**

The literature was searched through the PubMed, Web of Science, Cochrane Library, and Scopus databases from inception until September 2021.

**Data Extraction:**

Twenty-three eligible RCTs were initially identified. Ten RCTs examining the effect of creatine supplementation compared with placebo on measures of memory in healthy individuals met the inclusion criteria for systematic review, 8 of which were included in the meta-analysis.

**Data Analysis:**

Overall, creatine supplementation improved measures of memory compared with placebo (standard mean difference [SMD] = 0.29, 95%CI, 0.04–0.53; *I*^2^* *=* *66%; *P *=* *0.02). Subgroup analyses revealed a significant improvement in memory in older adults (66–76 years) (SMD = 0.88; 95%CI, 0.22–1.55; *I*^2^* *=* *83%; *P *=* *0.009) compared with their younger counterparts (11–31 years) (SMD = 0.03; 95%CI, −0.14 to 0.20; *I*^2^* *=* *0%; *P *=* *0.72). Creatine dose (≈ 2.2–20 g/d), duration of intervention (5 days to 24 weeks), sex, or geographical origin did not influence the findings.

**Conclusion:**

Creatine supplementation enhanced measures of memory performance in healthy individuals, especially in older adults (66–76 years).

**Systematic Review Registration:**

PROSPERO registration no. 42021281027.

## INTRODUCTION

The brain requires a high amount of energy for cellular processes, such as neurotransmitter exocytosis and synaptic functioning.^1^ Creatine, an organic acid obtained from the diet (primarily from red meat and seafood) or synthesized endogenously in the liver, the kidneys, and the brain,^2^ is an important molecule for energy production. Phosphocreatine and adenosine diphosphate are converted to creatine and adenosine triphosphate (ATP) in a reversible reaction catalyzed by creatine kinase.^2^ This conversion and production of ATP occurs faster than oxidative phosphorylation and glycolytic processes.^3^ Creatine supplementation increases brain creatine content and the ratio of phosphocreatine to ATP.^4^^,^^5^ Further, creatine attenuates reactive oxygen species via facilitation of mitochondrial ATP coupling or by scavenging radical species in an acellular setting.^6^

There is a growing body of literature on the neurobehavioral and physiological effects of creatine supplementation.^7–9^ Given that memory (defined as the ability to process and retain information) is energetically demanding and dependent on intact mitochondrial respiratory function,^10^ and considering that creatine is a key regulator of energy status,^2^ elevation of creatine levels in the brain may enhance memory by altering brain bioenergetics. In vitro, creatine elevates phosphocreatine and ATP levels^11^ and increases oxidative phosphorylation in synaptosomes and isolated brain mitochondria.^12^ In hippocampal neuron cultures, creatine stimulates mitochondrial activity.^13^ In rats, intrahippocampal injections of creatine in the CA1 subfield enhances spatial memory formation.^14^ Further, cAMP-response element binding protein (CREB), a key transcription factor involved in memory consolidation,^15^ is upregulated 30 minutes after creatine injection.^14^ More recently, Snow et al^16^ found that 4 weeks of creatine supplementation in mice increased coupled respiration in isolated hippocampal mitochondria and improved memory.

As an example of the importance of creatine in humans, creatine-deficient syndromes that deplete brain creatine stores are characterized by mental and developmental disorders such as learning delays and seizures^17^^,^^18^; importantly, these symptoms can be partially reversed by creatine supplementation.^19–21^ In healthy humans, there are mixed results: some studies reported benefits on cognitive functioning,^22–33^ while others found no effect.^34–36^ Likewise, the results of research regarding the effectiveness of creatine supplementation on improving measures of memory are mixed. Elderly study participants (68–85 years) who received creatine supplementation (20 g/d for 7 days) showed significant improvements in measures of memory (forward number recall, backward and forward spatial recall, and long-term memory) compared with those who received placebo.^25^ Similarly, Rae et al^37^ found improvements in working memory following creatine supplementation (5 g/d for 6 weeks) in vegetarians. In a direct comparison of omnivores and vegetarians, Benton and Donohoe^24^ found better memory following creatine supplementation (20 g/d for 5 days) in vegetarians compared with meat eaters. Nevertheless, this might be attributable to the intake of lower-creatine vegetarian diets.^38^^,^^39^ Other researchers, however, have failed to find beneficial effects of creatine supplementation on measures of memory in children,^36^ adults^27^^,^^28^^,^^30^^,^^34^ and older adults.^25^^,^^35^ While multifactorial, these inconsistent findings across individual studies may be related to methodological differences (dosage and duration of creatine supplementation), population characteristics (age, sex, geographical origin), or low sample size. Therefore, a systematic review and meta-analysis was performed to assess the effects of creatine supplementation vs placebo on memory performance in healthy humans.

## METHODS

This systematic review and meta-analysis was conducted in accordance with the PRISMA (Preferred Reporting Items for Systematic Reviews and Meta-Analyses) guidelines.^40^ The protocol was registered in the PROSPERO (International Prospective Register of Systematic Reviews) database (CRD:42021281027).

### Search strategy

Two independent reviewers (K.P. and P.G.) searched PubMed, Scopus, Web of Science, and the Cochrane library from inception until September 30, 2021. A search strategy involving the following terms was used: “creatine” OR “creatine monohydrate” AND “cogn*” OR “memory”. A manual search of references cited in the selected articles and published reviews was also performed. Discrepancies in the literature search process were resolved by a third investigator (K.S.K.). Studies were included according to the PICOS (Population, Intervention, Comparator, Outcomes, and Study design) process (Table 1). The following inclusion criteria were applied: (1) studies were randomized controlled trials (RCTs); (2) population comprised healthy participants; (3) intervention group received creatine supplementation; (4) control group received a placebo; and (5) memory performance outcomes were assessed. Studies were excluded if they were not RCTs; if a full text was not available; if participants with self-reported comorbidities were included; or if participants with any specific dietary restrictions (eg, vegetarians) were included.

**Table 1:** PICOS criteria for inclusion and exclusion of studies

Parameter	Inclusion criteria	Exclusion criteria
Population	Healthy adults	Populations with comorbidities or dietary restrictions (ie, vegan/vegetarians)
Intervention	Creatine monohydrate supplementation	Other additional nutritional interventions
Comparator	Placebo	Non-placebo control
Outcomes	Memory performance outcomes	Other outcomes related to cognitive function
Study design	Randomized controlled trials	Non-randomized controlled trials, cohort studies, in vivo studies, in vitro studies

### Data extraction and risk of bias

Two authors (K.P. and P.G.) extracted data independently. Extracted data included name of first author; date of publication; study design; age, sex, and health status of participants, number of participants; outcomes measured; and form, dose, and duration of treatment. Disagreements between authors were resolved by a third reviewer (K.K.T.). The quality of included studies was assessed using version 2 of the Cochrane risk-of-bias 2 tool for randomized trials (RoB 2) and evaluated by 3 independent reviewers (K.P., P.G., and K.K.T.). Appraisal of risk of bias using the RoB 2 tool included the assessment of the following domains of bias in RCTs: (1) randomization process, (2) deviations from intended interventions, (3) missing outcome data, (4) measurement of the outcome, and (5) selection of the reported result. In accordance with the RoB 2 tool scoring system, study quality was defined as low risk of bias, some concerns, or high risk of bias.

### Outcome assessment

Memory performance was considered the main outcome in the analysis and comprised multiple measures of memory from comparable studies, with no restrictions placed on the tool used for assessment.

### Statistical analysis

The meta-analysis compared changes in memory performance in participants who received either creatine monohydrate or placebo. Quantitative data were treated as continuous measurements, and changes in outcomes from baseline to follow-up were compared between groups to calculate mean differences. When units of measurements were inconsistent and could not be converted to units required to be included in the analysis or when different aspects of memory were measured as outcomes, standardized mean differences were used. Statistical significance was assessed using the random-effects model and the inverse-variance method. Any changes between baseline and follow-up outcome measurements for which standard deviations were missing were estimated by calculating a correlation coefficient from a known change from the baseline standard deviation derived from a similar study.

Statistical heterogeneity of outcome measurements between different studies was assessed using the overlap of their confidence intervals (95%CIs) and expressed as a measurement of Cochran's Q (χ^2^ test) and *I*^2^. Data were classified as moderately heterogeneous when *I*^2^ values ranged from 50% to 4.9% and as highly heterogeneous when values were 75% and above. Furthermore, sensitivity analysis was performed to evaluate the robustness of reported statistical results by discounting the effects of creatine form (encapsulated vs powder), conditions of stress (normal vs stressed [following hypoxia, sleep deprivation, or exhaustive exercise]), and rate of participants lost during follow-up (< 15% loss vs ≥ 15% loss). Subgroup analyses based on age (< 50 years vs ≥ 50 years), sex (males only vs females only vs mixed sexes), treatment duration (< 2 weeks vs ≥ 2 weeks), and dose (low dose ≤ 5 g/d vs high dose > 5 g/d) of creatine monohydrate supplementation were also performed. The meta-analysis of data was synthesized using Cochrane’s Review Manager (RevMan 5.4.1) software.

## RESULTS

**Figure 1:**
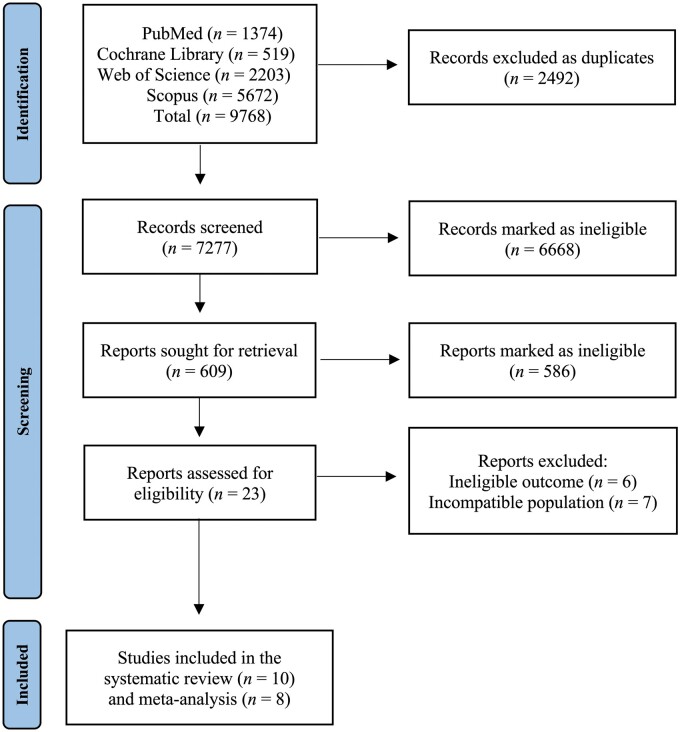
Flow diagram of the literature search process.

Figure 1 shows the literature search process. The initial literature search yielded 9768 publications. After exclusion of 2492 duplicates, 7277 unique publications were identified. Following screening of titles and abstracts, 6668 publications with irrelevant study design were excluded and 609 RCTs were retrieved. Further screening of abstracts of the remaining publications resulted in 23 eligible RCTs examining the effects of creatine supplementation on performance measures of memory. Of these, 6 studies had ineligible outcomes and 7 had incompatible study populations. Ten studies were included in the systematic review^22^^,^^24^^,^^25^^,^^27^^,^^28^^,^^30^^,^^33–36^ and 8 in the meta-analysis^25^^,^^27^^,^^28^^,^^30^^,^^33–36^; 2 studies were excluded from the meta-analysis because they were missing baseline outcomes^24^ or standard deviations of outcomes.^22^

Of the 10 studies, 4 were conducted in the United Kingdom,^24^^,^^25^^,^^27^^,^^28^ 4 in Brazil,^22^^,^^33^^,^^35^^,^^36^ 1 in New Zealand,^30^ and 1 in the United States.^34^ Eight were conducted in young adults^22^^,^^24^^,^^27^^,^^28^^,^^30^^,^^33^^,^^34^^,^^36^ and 2 in older adults.^25^^,^^35^ Five studies were conducted in cohorts of both males and females,^27^^,^^28^^,^^30^^,^^34^^,^^36^ 3 in females only,^24^^,^^33^^,^^35^ and 2 in males only.^22^^,^^27^ The duration of creatine supplementation was 5 days in 1 study,^24^ 7 days in 5 studies,^22^^,^^25^^,^^27^^,^^28^^,^^36^ 2 weeks in 1 study,^30^ 4 weeks in 1 study,^33^ 6 weeks in 1 study,^34^ and 24 weeks in 1 study.^35^ The daily dose of creatine was 20 g in 7 studies,^22^^,^^24^^,^^25^^,^^27^^,^^28^^,^^30^ 5 g in 1 study,^35^ and 3 g in 1 study.^33^ One study supplemented with a dosage of 0.3 g/kg/d divided into 4 doses, which equated to a mean absolute dose of 13.7 g/d,^36^ while another study supplemented with a dosage of 0.03 g/kg/d, which was equivalent to approximately 2.2 g/d.^34^ Nine studies were double-blind RCTs,^22^^,^^24^^,^^25^^,^^27^^,^^28^^,^^33–36^ while 1 study was a double-blind crossover RCT.^30^ Nine studies used creatine monohydrate in powder form,^22^^,^^24^^,^^25^^,^^27^^,^^28^^,^^30^^,^^33^^,^^35^^,^^36^ while 1 study used creatine in encapsulated form.^34^ Overall, 8 studies with a total of 225 participants (74 males and 151 females; 122 in creatine group, 118 in placebo group) were included in the meta-analysis (Table 2).^25^^,^^27^^,^^28^^,^^30^^,^^33–36^

**Table 2:** Characteristics of the studies included in the meta-analysis

Reference	Total, both groups		Creatine group		Placebo group		Treatment dosage (duration)	Memory outcomes
	No. (M/F)	Age of participants	No. (M/F)	Age of participants	No. (M/F)	Age of participants		
Alves et al (2013)^35^	25 (0/25)	60–80 y	13 (0/13)	62–72 y	12 (0/12)	61–73 y	5 g/d (24 wk)	BBCS (delayed recall, immediate memory, incidental memory, learning, naming)
McMorris et al (2006)^28^	19 (16/3)	19–23 y	10 (9/1)	NA	9 (7/2)	NA	20 g/d (7 d)	Backward spatial recall, forward spatial recall, backward verbal recall, forward verbal recall
McMorris et al (2007a)^27^	19 (19/0)	19–23 y	10 (10/0)	NA	9 (9/0)	NA	20 g/d (7 d)	Forward number recall
McMorris et al (2007b)^25^	32 (16/16)	68–85 y	15 (8/7)	NA	17 (8/9)	NA	20 g/d (1 wk)	Forward number recall, backward number recall, backward spatial recall, forward spatial recall, long-term memory
Merege-Filho et al (2017)^36^	67 (0/67)	10–12 y	35 (19/16)	11–13 y	32 (19/13)	11–13 y	13.7 g/d (7 d)	RAVLT (learning, short-term memory, long-term memory)
Pires et al (2020)^33^	26 (0/26)	21–31 y	13 (0/13)	21–31 y	13 (0/13)	21–31 y	3 g/d (28 d)	Corsi block test, differentiation task test, reverse Corsi block test, visual forward digit span test
Rawson et al (2008)^34^	22 (13/9)	19–23 y	11 (6/5)	19–23 y	11 (6/5)	19–23 y	2.2 g/d (6 wk)	Memory recall (correct, all, throughput)
Turner et al (20115)^30^	15 (20/10)	21–55 y	15 (10/5)	21–55 y	15 (10/5)	21–55 y	20 g/d (7 d)	Composite memory

*Abbreviations*: BBCS, Brief Battery of Cognitive Screening; NA, not available; RAVLT, Rey Auditory Verbal Learning Test.

### Definition of memory outcomes

Memory was evaluated through multiple assessment tools in the included studies. The Rey Auditory Verbal Learning Test is comprised of 4 tests assessing free and delayed recall memory immediately after and following 20 minutes of a 12-word list presentation, respectively.^36^ A digit span test was used to assess short-term memory by asking participants to repeat a sequence of digits forwards and backwards.^33^^,^^35^ In this test, performance assessment was based on the number of digits participants were able to recall correctly. Further, running memory was evaluated through the correct guess of participants pressing a specified mouse button key that would match an immediate letter shown on the screen for 1 second.^34^ For the Sternberg memory task, participants were asked to memorize a set of 6 letters displayed on a monitor screen for 20 seconds.^34^ Thereafter, letters were presented on the screen one at a time, and participants had to press a specific mouse button to indicate whether the screen letter was present in the memorized set. Definitions of measurements used to assess aspects of memory and composite memory through the Brief Battery of Cognitive Screening were not provided. The Corsi block test, a variation of the Corsi block tapping test, was employed to assess memory recall and reproduction of block position sequences in a screen.^33^ Furthermore, the reverse Corsi block test was used to reproduce the sequences in reverse.^33^ The visual forward digit span test was utilized to evaluate short-term memory through a phonological loop, aiming at the recall of as many digits as possible.^33^

### Creatine supplementation and memory performance

**Figure 2:**
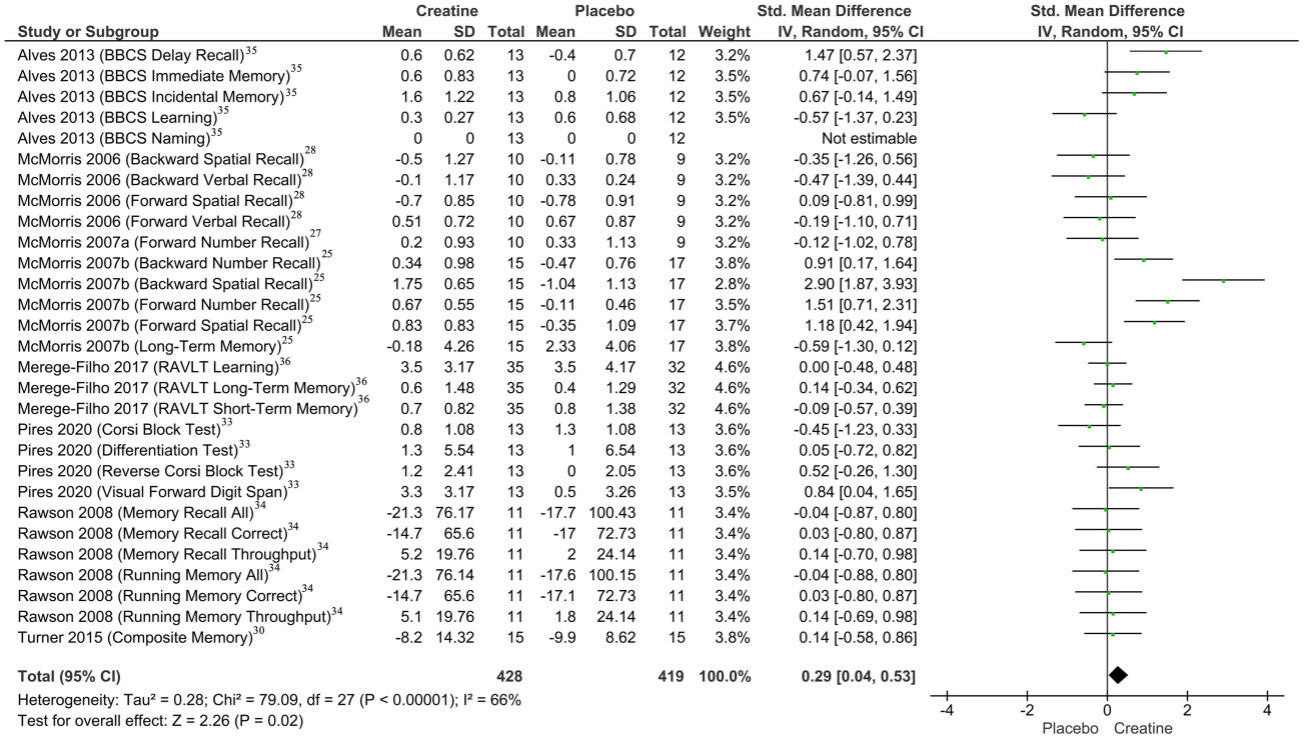
Effect of creatine monohydrate supplementation on overall memory.

The main analysis showed that memory performance following creatine supplementation, compared with placebo, was improved, although a moderate degree of heterogeneity was observed between the included RCTs (standard mean difference [SMD] = 0.29; 95%CI, 0.04–0.53; *I*^2^* *=* *66%; *P *=* *0.02) (Figure 2).^25^^,^^27^^,^^28^^,^^30^^,^^33–36^

**Figure 3:**
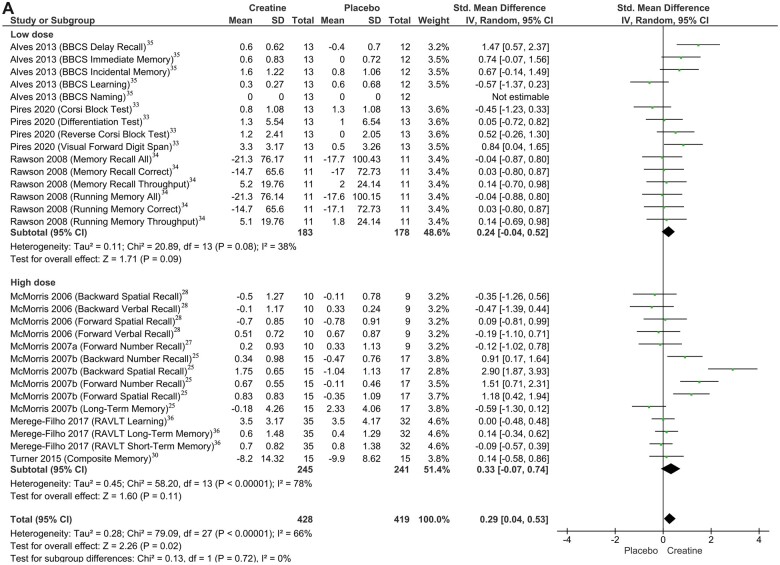

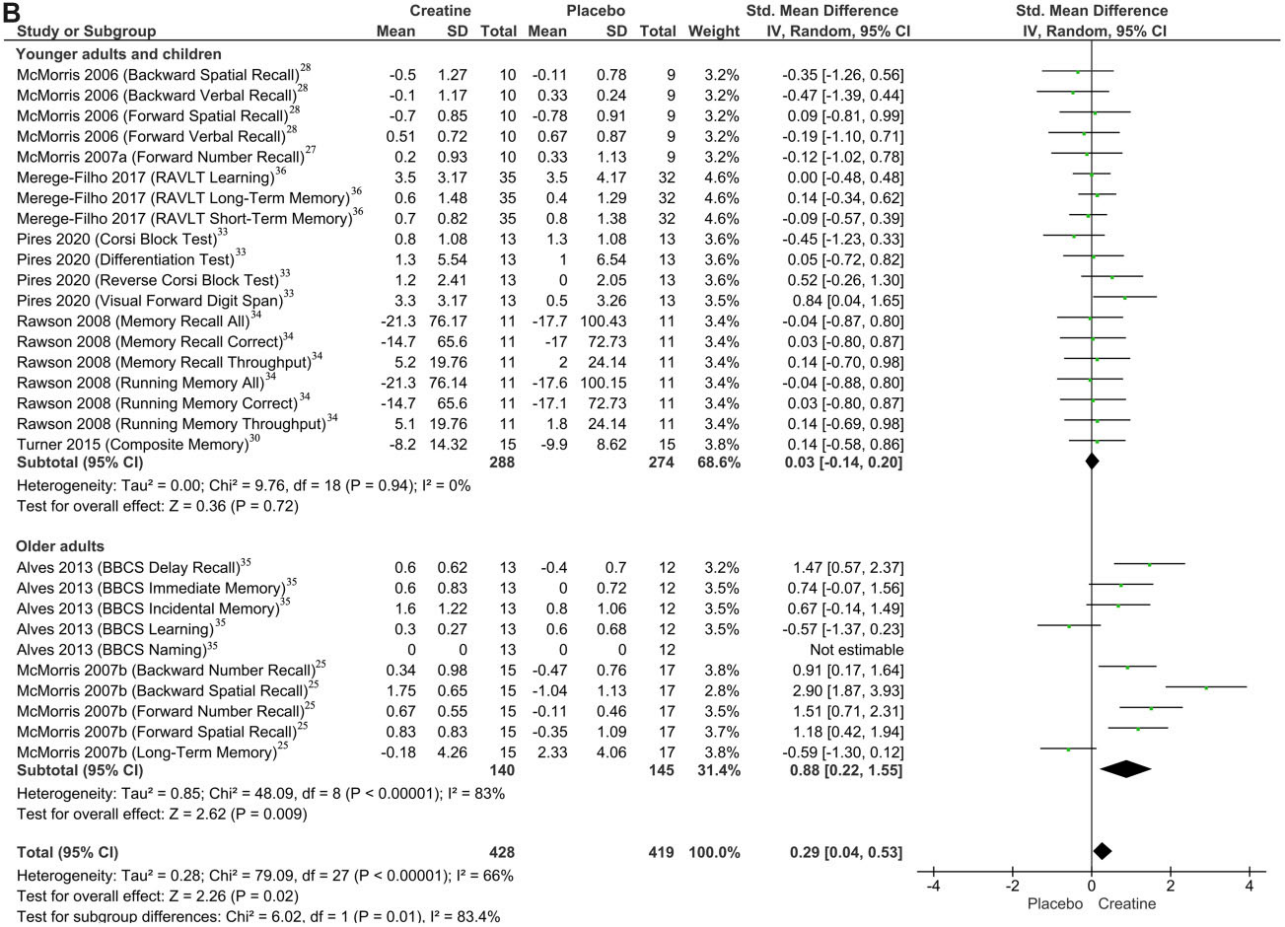

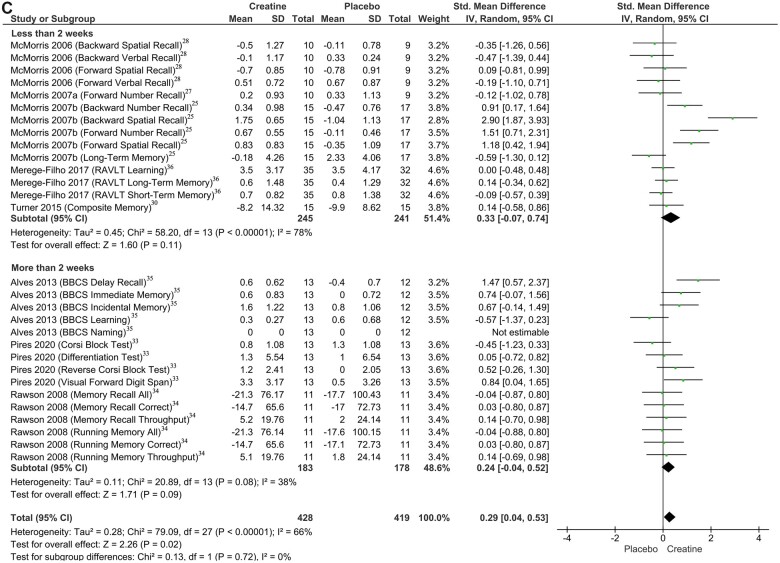

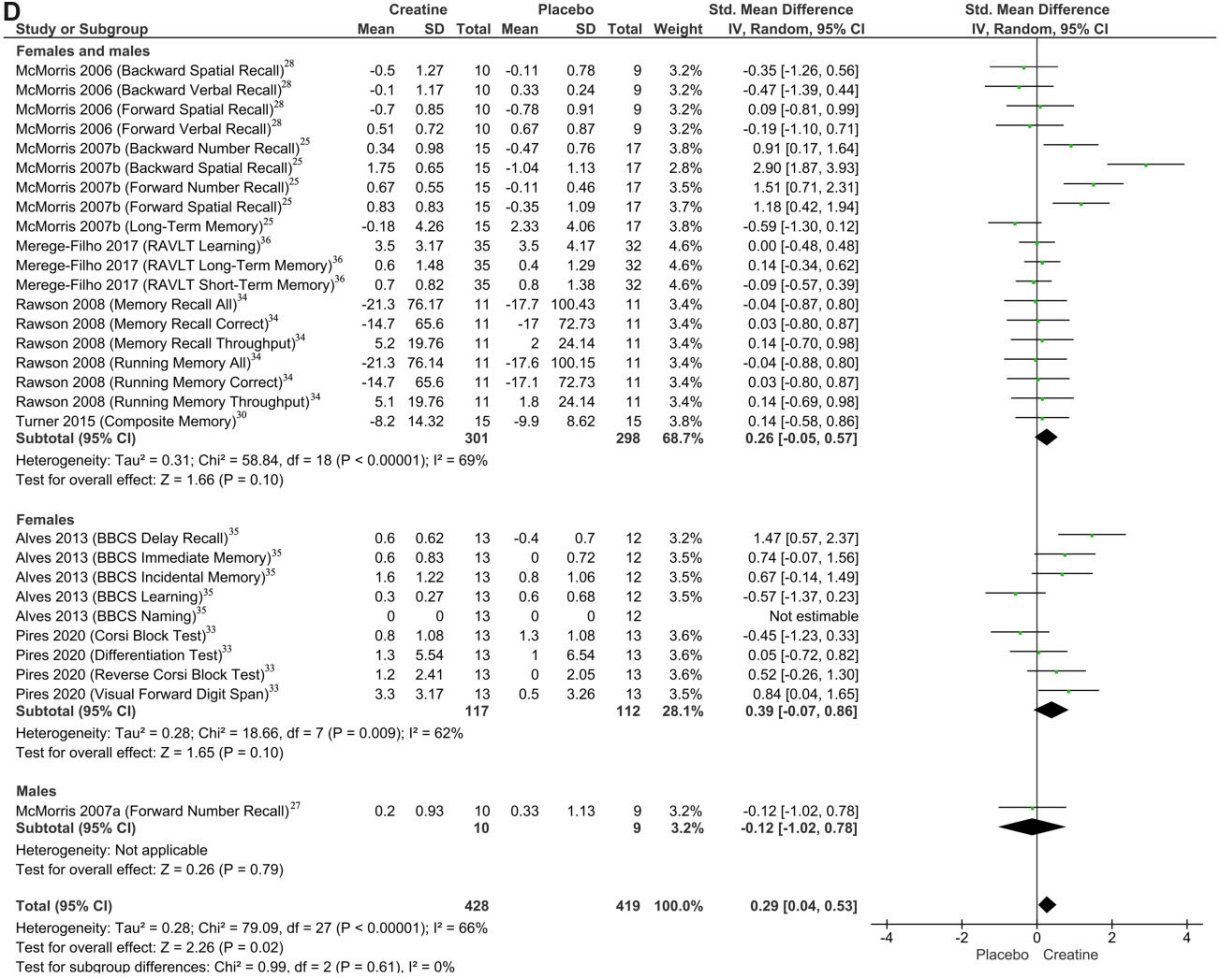
Subgroup analysis of the effect of creatine monohydrate supplementation on overall memory, based on (A) dose of creatine monohydrate, (B) age of individual, (C) duration of supplementation, and (D) sex of individual.

A series of subgroup analyses showed that creatine monohydrate supplementation in low doses (≤ 5 g/d) (SMD = 0.24; 95%CI, −0.04 to 0.52; *I*^2^* *=* *38%; *P *=* *0.09) or high doses (> 5 g/d) (SMD = 0.33; 95%CI; −0.07 to 0.74; *I*^2^* *=* *78%; *P *=* *0.11) was not associated with improvements in memory measures (Figure 3A).^25^^,^^27^^,^^28^^,^^30^^,^^33–36^ Additionally, no differences following supplementation were observed in young adults (11–31 years) (SMD = 0.03; 95%CI, −0.14 to 0.20; *I*^2^* *=* *0%; *P *=* *0.72); however, in older adults (66–76 years), increased memory performance was observed (SMD = 0.88; 95%CI, 0.22–1.55; *I*^2^* *=* *83%; *P *=* *0.009) (Figure 3B).^25^^,^^27^^,^^28^^,^^30^^,^^33–36^ Outcome measures were also not affected by treatment duration (≤ 2 weeks, SMD = 0.33; 95%CI, −0.07 to 0.74; *I*^2^* *=* *78%; *P *=* *0.11; > 2 weeks, SMD = 0.24; 95%CI, −0.04 to 0.52; *I*^2^* *=* *38%; *P *=* *0.09) (Figure 3C)^25^^,^^27^^,^^28^^,^^30^^,^^33–36^ or sex ([females and males, SMD = 0.26; 95%CI, −0.05 to 0.57; *I*^2^* *=* *69%; *P *=* *0.10], [females, SMD = 0.39; 95%CI, −0.07 to 0.86; *I*^2^* *=* *62%; *P *=* *0.10], [males, SMD = −0.12; 95%CI, −1.02 to 0.78; *P *=* *0.79]) (Figure 3D).^25^^,^^27^^,^^28^^,^^30^^,^^33–36^

A significant improvement in memory measures following supplementation with creatine monohydrate in powder form was observed (SMD = 0.35; 95%CI, 0.05–0.66; *I*^2^* *=* *73%; *P *=* *0.02), but no effect of encapsulated creatine monohydrate was found (SMD = 0.04; 95%CI, −0.30 to 0.39; *I*^2^* *=* *0%; *P *=* *0.80) (see Figure S1A in the Supporting Information online). Additionally, outcome measures were significantly improved under nonstressed conditions (SMD = 0.43; 95%CI, 0.09–0.78; *I*^2^* *=* *75%; *P *=* *0.01) but remained unchanged under stressed conditions (SMD = 0.03; 95%CI, −0.23 to 0.30; *I*^2^* *=* *1%; *P *=* *0.80) (see Figure S1B in the Supporting Information online). Furthermore, in cohorts in which loss to follow-up was below 15%, significant improvements in memory were seen (SMD = 0.33; 95%CI, 0.04–0.62; *I*^2^* *=* *68%; *P *=* *0.03), while no significant differences were observed in cohorts in which the rate exceeded 15% (SMD = 0.02; 95%CI, −0.26 to 0.29; *I*^2^* *=* *0%; *P *=* *0.90) (see Figure S1C in the Supporting Information online).

### Assessment of risk of bias and quality of evidence

Risk of bias was scored as high in 6 RCTs because information about how randomization was applied was lacking.^22^^,^^24^^,^^25^^,^^27^^,^^28^^,^^30^ In 4 RCTs, some concerns were raised because of the absence of detail regarding treatment allocation.^22^^,^^24^^,^^30^^,^^34^ In 1 RCT, some concern was recorded because the number of participants who dropped out and the reasons for withdrawing were poorly defined.^24^ Finally, 8 RCTs had a study protocol that was not prespecified, resulting in some concerns.^22^^,^^24^^,^^25^^,^^27^^,^^28^^,^^30^^,^^34^^,^^36^ A detailed traffic light plot showing the results of quality assessment of the included studies is presented in Figure S2 in the Supporting Information online.

## DISCUSSION

This is the first meta-analysis to examine the effectiveness of creatine supplementation on memory performance in healthy individuals. Results showed that measures of memory following creatine supplementation, compared with placebo, were improved. These benefits were more robust in older adults (66–76 years). However, a moderate risk of bias and significant heterogeneity between studies were observed, and therefore caution is warranted.

These beneficial effects from creatine supplementation on memory performance may be related to creatine’s ability to influence brain bioenergetics. For example, creatine elevates phosphocreatine and ATP levels^11^ and increases oxidative phosphorylation in synaptosomes and isolated brain mitochondria.^12^ In hippocampal neuron cultures, creatine stimulates mitochondrial activity.^13^ In animal models, intrahippocampal injections of creatine in the CA1 subfield enhances spatial memory formation in the Barnes maze in rats and in the object exploration task in mice.^14^ Further, CREB, a key transcription factor that is well established in activity-dependent plasticity, learning, and memory,^15^ is upregulated 30 minutes after creatine injection.^14^ Snow et al^16^ found that 4 weeks of creatine supplementation in mice increased coupled respiration in isolated hippocampal mitochondria and improved memory. Moreover, creatine supplementation has been shown to elevate brain creatine content and the ratio of phosphocreatine to ATP in humans.^4^^,^^5^ Not all studies, however, found these increases,^36^ possibly owing to limited creatine transporters at the blood-brain barrier or the ability of the brain to synthesize creatine.^7^ Additionally, brain creatine content may decline during aging.^41^ In theory, those who have lower levels of creatine in the brain may be more responsive to creatine supplementation,^42^^,^^43^ which is similar to what has been observed in muscle.^44^ The subgroup analysis based on age revealed a greater effect size following creatine supplementation in older adults (66–76 years) as opposed to younger individuals (11–31 years). These findings may be clinically important and further highlight the need for additional clinical research to determine the mechanistic actions of creatine in large cohorts of healthy older adults and those with neurological and neurodegenerative diseases in whom brain creatine levels and memory are compromised.

This meta-analysis also reveals that supplementation with creatine monohydrate had no influence on memory performance following a higher dosing strategy (ie, > 5 g/d). Although there are limited data exploring the effect of dose-response relationships of creatine on memory, the results suggest that the amount of either endogenous creatine synthesis or dietary creatine intake may be sufficient to maintain adequate brain creatine stores^45^ and that a higher exogenous dose of creatine is not required, despite the limited ability of creatine to cross the blood-brain barrier. As such, higher doses of creatine supplementation may not be required to optimize brain creatine content^4^^,^^5^ and ATP (re)synthesis by mitochondrial creatine kinases.^46^ Presently, very little is known about the effect of the duration of the intervention. The subgroup analysis revealed no significant differences between short-term (≤ 2 weeks) and long-term (> 2 weeks) studies, but it is likely that much-longer-term trials are required to show robust changes over time.

Another potential mediating factor is dietary intake of creatine.^39^ Presently there are very few studies directly comparing vegetarians with omnivores.^24^ Vegetarians have a lower creatine content in muscle and are more responsive to creatine supplementation.^38^ A previous RCT found that word recall response declined following creatine supplementation (20 g/d of creatine for 5 days) in meat-eaters compared with vegan and vegetarian participants, and post-supplementation comparisons revealed a significantly greater memory in vegetarians compared with omnivores.^24^ Despite lower creatine muscle content, Solis et al^47^ did not find any differences between vegetarians and omnivores with regard to brain creatine content. Future research is warranted to confirm these findings.

### Strengths and limitations

This is the first meta-analysis to examine the effects of creatine monohydrate supplementation on memory performance in healthy individuals and to determine whether quantifiable differences in exogenous supplementation based on dose, treatment duration, and age modulate measures of memory. A limitation, however, is that the included studies did not assess baseline levels of serum or brain creatine. As such, it was not possible to determine whether non-responders had lower brain creatine levels than responders. Therefore, it remains inconclusive if the differential effects of creatine monohydrate supplementation between responders and non-responders are directly linked to discrepancies in baseline creatine levels or a metabolic dimorphism in any specific mechanism of creatine monohydrate. There are also several methodological limitations, including the combination of various assessment tools designed to measure memory, which increases the heterogeneity of the data. In particular, several memory tests were combined to increase the statistical power (including short-term, long-term, and working memory). For example, backward number recall, which may require greater energy production than forward recall, may generate a stronger activation of the parietal, occipital, frontal, and temporal cortices, as shown in functional magnetic resonance imaging scans.^48^ Presently, it is unclear if there are regional differences in the uptake and utilization—and, thus, the effects—of creatine supplementation on cognitive function. Additionally, some memory tasks required an initial learning phase that likely employs auxiliary aspects of cognition, such as attention. Thus, it is likely that a proportion of these tests may have exceeded a specific difficulty threshold. Furthermore, most of the included studies did not assess creatine intake from dietary consumption, which may have altered the findings.^24^

The findings presented here should be interpreted with caution. The included studies were of moderate quality, according to the Cochrane RoB 2 tool. This is likely attributable in part to the presence of confounders, the inherent heterogeneity between intervention and placebo groups, and the suboptimal selection of participants in the included studies. Moreover, the high diversity between the memory assessment tools could also influence the accuracy and lead to uncertainty of the effect estimate. Importantly, subgroup and sensitivity analyses in this study were not able to reduce this heterogeneity, especially since different memory outcomes were derived from the same populations in each study. Finally, it was not possible to assess publication bias, which could affect the quality of evidence, because the number of included studies was too low.

## CONCLUSION

This systematic review and meta-analysis revealed that creatine monohydrate supplementation has a beneficial effect on memory performance in healthy individuals. Subgroup analysis showed the effects of creatine were more robust in older adults. The lack of homogeneity in outcomes of memory performance illustrates an unmet demand for common assessment tools that could be used by both researchers and practitioners in the pursuit of results with higher precision and accuracy. As such, future research utilizing a rigorous, large, long-term randomized clinical trial to elucidate the potential effect of creatine monohydrate supplementation on memory performance is urgently warranted.

## Supplementary Material

nuac064_Supplementary_Data

## References

[1] Jaumann S , Scudelari R, Naug D. Energetic cost of learning and memory can cause cognitive impairment in honeybees. Biol Lett. 2013;9:20130149. 23784929 10.1098/rsbl.2013.0149 PMC3730623

[2] Wyss M , Kaddurah-Daouk R. Creatine and creatinine metabolism. Physiol Rev. 2000;80:1107–1213. 10893433 10.1152/physrev.2000.80.3.1107

[3] Wallimann T , Wyss M, Brdiczka D, et al Intracellular compartmentation, structure and function of creatine kinase isoenzymes in tissues with high and fluctuating energy demands: the ‘phosphocreatine circuit’ for cellular energy homeostasis. Biochem J. 1992;281:21–40. 1731757 10.1042/bj2810021 PMC1130636

[4] Dechent P , Pouwels P, Wilken B, et al Increase of total creatine in human brain after oral supplementation of creatine-monohydrate. Am J Physiol Regulat Integr Comparat Physiol. 1999;277:R698–R704. 10.1152/ajpregu.1999.277.3.R698 10484486

[5] Pan JW , Takahashi K. Cerebral energetic effects of creatine supplementation in humans. Am J Physiol Regulat Integr Comparat Physiol. 2007;292:R1745–R1750. 10.1152/ajpregu.00717.2006 PMC3570026 17185404

[6] Sestili P , Martinelli C, Colombo E, et al Creatine as an antioxidant. Amino Acids. 2011;40:1385–1396. 21404063 10.1007/s00726-011-0875-5

[7] Roschel H , Gualano B, Ostojic SM, et al Creatine supplementation and brain health. Nutrients. 2021;13:586. 33578876 10.3390/nu13020586 PMC7916590

[8] Forbes SC , Candow DG, Ferreira LH, et al Effects of creatine supplementation on properties of muscle, bone, and brain function in older adults: a narrative review. J Diet Suppl. 2022;19:318–335. 33502271 10.1080/19390211.2021.1877232

[9] Forbes SC , Cordingley DM, Cornish SM, et al Effects of creatine supplementation on brain function and health. Nutrients. 2022;14:921. 35267907 10.3390/nu14050921 PMC8912287

[10] Tanaka D , Nakada K, Takao K, et al Normal mitochondrial respiratory function is essential for spatial remote memory in mice. Mol Brain. 2008;1:21–16. 19087269 10.1186/1756-6606-1-21 PMC2653021

[11] Brewer GJ , Wallimann TW. Protective effect of the energy precursor creatine against toxicity of glutamate and β‐amyloid in rat hippocampal neurons. J Neurochem. 2000;74:1968–1978. 10800940 10.1046/j.1471-4159.2000.0741968.x

[12] Monge C , Beraud N, Kuznetsov AV, et al Regulation of respiration in brain mitochondria and synaptosomes: restrictions of ADP diffusion in situ, roles of tubulin, and mitochondrial creatine kinase. Mol Cell Biochem. 2008;318:147–165. 18629616 10.1007/s11010-008-9865-7

[13] Li Z , Okamoto K-I, Hayashi Y, et al The importance of dendritic mitochondria in the morphogenesis and plasticity of spines and synapses. Cell. 2004;119:873–887. 15607982 10.1016/j.cell.2004.11.003

[14] Souza MA , Magni DV, Guerra GP, et al Involvement of hippocampal CAMKII/CREB signaling in the spatial memory retention induced by creatine. Amino Acids. 2012;43:2491–2503. 22669403 10.1007/s00726-012-1329-4

[15] Alberini CM , Kandel ER. The regulation of transcription in memory consolidation. Cold Spring Harb Perspect Biol. 2014;7:a021741. 25475090 10.1101/cshperspect.a021741 PMC4292167

[16] Snow WM , Cadonic C, Cortes-Perez C, et al Chronic dietary creatine enhances hippocampal-dependent spatial memory, bioenergetics, and levels of plasticity-related proteins associated with NF-κB. Learn Mem. 2018;25:54–66. 29339557 10.1101/lm.046284.117 PMC5772392

[17] Bender A , Klopstock T. Creatine for neuroprotection in neurodegenerative disease: end of story? Amino Acids. 2016;48:1929–1940. 26748651 10.1007/s00726-015-2165-0

[18] Mercimek-Andrews S , Salomons GS. Creatine deficiency disorders. In: Adam MP, Mirzaa GM, Pagon RA, et al, eds. Gene Reviews [internet]. University of Washington; 2009. https://www.ncbi.nlm.nih.gov/books/NBK3794/20301745

[19] Kaldis P , Hemmer W, Zanolla E, et al ‘Hot spots’ of creatine kinase localization in brain: cerebellum, hippocampus and choroid plexus. Dev Neurosci. 1996;18:542–554. 8940630 10.1159/000111452

[20] Salomons G , Van Dooren S, Verhoeven N, et al X‐linked creatine transporter defect: an overview. J Inherit Metab Dis. 2003;26:309–318. 12889669 10.1023/a:1024405821638

[21] Stöckler S , Holzbach U, Hanefeld F, et al Creatine deficiency in the brain: a new, treatable inborn error of metabolism. Pediatr Res. 1994;36:409–413. 7808840 10.1203/00006450-199409000-00023

[22] Borchio L , Machek SB, Machado M. Supplemental creatine monohydrate loading improves cognitive function in experienced mountain bikers. J Sports Med Phys Fitness. 2020;60:1168–1170. 32955844 10.23736/S0022-4707.20.10589-9

[23] Hammett ST , Wall MB, Edwards TC, et al Dietary supplementation of creatine monohydrate reduces the human fMRI BOLD signal. Neurosci Lett. 2010;479:201–205. 20570601 10.1016/j.neulet.2010.05.054

[24] Benton D , Donohoe R. The influence of creatine supplementation on the cognitive functioning of vegetarians and omnivores. Br J Nutr. 2011;105:1100–1105. 21118604 10.1017/S0007114510004733

[25] McMorris T , Mielcarz G, Harris RC, et al Creatine supplementation and cognitive performance in elderly individuals. Neuropsychol Dev Cogn B Aging Neuropsychol Cogn. 2007;14:517–528. 17828627 10.1080/13825580600788100

[26] Ling J , Kritikos M, Tiplady B. Cognitive effects of creatine ethyl ester supplementation. Behav Pharmacol. 2009;20:673–679. 19773644 10.1097/FBP.0b013e3283323c2a

[27] McMorris T , Harris RC, Howard AN, et al Creatine supplementation, sleep deprivation, cortisol, melatonin and behavior. Physiol Behav. 2007;90:21–28. 17046034 10.1016/j.physbeh.2006.08.024

[28] McMorris T , Harris RC, Swain J, et al Effect of creatine supplementation and sleep deprivation, with mild exercise, on cognitive and psychomotor performance, mood state, and plasma concentrations of catecholamines and cortisol. Psychopharmacology (Berl). 2006;185:93–103. 16416332 10.1007/s00213-005-0269-z

[29] Smolarek AC , McAnulty SR, Ferreira LH, et al Effect of 16 weeks of strength training and creatine supplementation on strength and cognition in older adults: a pilot study. J Exerc Physiol Online. 2020;23:88–95.

[30] Turner CE , Byblow WD, Gant N. Creatine supplementation enhances corticomotor excitability and cognitive performance during oxygen deprivation. J Neurosci. 2015;35:1773–1780. 25632150 10.1523/JNEUROSCI.3113-14.2015 PMC6795258

[31] Van Cutsem J, Roelands B, Pluym B, et al Can creatine combat the mental fatigue-associated decrease in visuomotor skills? *Med Sci Sports Exer*. 2020;52:120–130. 10.1249/MSS.0000000000002122 31403610

[32] Watanabe A , Kato N, Kato T. Effects of creatine on mental fatigue and cerebral hemoglobin oxygenation. Neurosci Res. 2002;42:279–285. 11985880 10.1016/s0168-0102(02)00007-x

[33] Pires LAM , Forbes SC, Candow DG, et al Creatine supplementation on cognitive performance following exercise in female Muay Thai athletes. NeuroSports. 2020;1:6.

[34] Rawson ES , Lieberman HR, Walsh TM, et al Creatine supplementation does not improve cognitive function in young adults. Physiol Behav. 2008;95:130–134. 18579168 10.1016/j.physbeh.2008.05.009

[35] Alves CRR , Merege Filho CAA, Benatti FB, et al Creatine supplementation associated or not with strength training upon emotional and cognitive measures in older women: a randomized double-blind study. PLoS One. 2013;8: E 76301. 10.1371/journal.pone.0076301 PMC3789718 24098469

[36] Merege-Filho CAA , Otaduy MCG, de Sá-Pinto AL, et al Does brain creatine content rely on exogenous creatine in healthy youth? A proof-of-principle study. Appl Physiol Nutr Metab. 2017;42:128–134. 28079396 10.1139/apnm-2016-0406

[37] Rae C , Digney AL, McEwan SR, et al Oral creatine monohydrate supplementation improves brain performance: a double-blind, placebo-controlled, cross-over trial. Proc R Soc Lond B. 2003;270:2147–2150. 10.1098/rspb.2003.2492 PMC1691485 14561278

[38] Burke DG , Chilibeck PD, Parise G, et al Effect of creatine and weight training on muscle creatine and performance in vegetarians. Med Sci Sports Exerc 2003;35:1946–1955. 10.1249/01.MSS.0000093614.17517.79 14600563

[39] Kaviani M , Shaw K, Chilibeck PD. Benefits of creatine supplementation for vegetarians compared to omnivorous athletes: a systematic review. Int J Environ Res Public Health. 2020;17:3041. 32349356 10.3390/ijerph17093041 PMC7246861

[40] Page MJ , McKenzie JE, Bossuyt PM, et al The PRISMA 2020 statement: an updated guideline for reporting systematic reviews. Syst Rev. 2021;10:89. 33781348 10.1186/s13643-021-01626-4 PMC8008539

[41] Laakso M , Hiltunen Y, Könönen M, et al Decreased brain creatine levels in elderly apolipoprotein E ε4 carriers. J Neural Transm (Vienna). 2003;110:267–275. 12658375 10.1007/s00702-002-0783-7

[42] Seper V , Korovljev D, Todorovic N, et al Guanidinoacetate-creatine supplementation improves functional performance and muscle and brain bioenergetics in the elderly: a pilot study. Ann Nutr Metab. 2021;77:244–247. 34515048 10.1159/000518499

[43] Balestrino M , Adriano E. Beyond sports: efficacy and safety of creatine supplementation in pathological or paraphysiological conditions of brain and muscle. Med Res Rev. 2019;39:2427–2459. 31012130 10.1002/med.21590

[44] Syrotuik DG , Bell GJ. Acute creatine monohydrate supplementation: a descriptive physiological profile of responders vs. nonresponders. J Strength Cond Res 2004;18:610–617. 10.1519/12392.1 15320650

[45] Brosnan ME , Brosnan JT. The role of dietary creatine. Amino Acids. 2016;48:1785–1791. 26874700 10.1007/s00726-016-2188-1

[46] Saks V , Kongas O, Vendelin M, et al Role of the creatine/phosphocreatine system in the regulation of mitochondrial respiration. Acta Physiol Scand. 2000;168:635–641. 10759600 10.1046/j.1365-201x.2000.00715.x

[47] Solis MY , Artioli GG, Otaduy MCG, et al Effect of age, diet, and tissue type on PCr response to creatine supplementation. J Appl Physiol. 2017;123:407–414. 28572496 10.1152/japplphysiol.00248.2017

[48] Sun X , Zhang X, Chen X, et al Age-dependent brain activation during forward and backward digit recall revealed by fMRI. Neuroimage. 2005;26:36–47. 15862203 10.1016/j.neuroimage.2005.01.022

