# Supplementary material for: Effects of creatine supplementation on memory in healthy individuals: a systematic review and meta-analysis of randomized controlled trials
Source: Nutr Rev. 2022 Aug 19;81(4):416–27. doi: 10.1093/nutrit/nuac064 (PMC9999677; doi:10.1093/nutrit/nuac064)
Supplement: nuac064_Supplementary_Data [file nuac064_supplementary_data.zip › Supporting_Information_References.docx]

**SUPPORTING INFORMATION**

**REFERENCES**

S1. Alves CRR, Merege Filho CAA, Benatti FB, et al. Creatine supplementation associated or not with strength training upon emotional and cognitive measures in older women: a randomized double-blind study. *PLoS One*. 2013;8(10):e76301.

S2. McMorris T, Harris RC, Swain J, et al. Effect of creatine supplementation and sleep deprivation, with mild exercise, on cognitive and psychomotor performance, mood state, and plasma concentrations of catecholamines and cortisol. *Psychopharmacology.* 2006;185(1):93-103.

S3. McMorris T, Harris RC, Howard AN, et al. Creatine supplementation, sleep deprivation, cortisol, melatonin and behavior. *Physiology & behavior*. 2007;90(1):21-28.

S4. McMorris T, Mielcarz G, Harris RC, Swain JP, Howard A. Creatine supplementation and cognitive performance in elderly individuals. *Aging, Neuropsychology, and Cognition*. 2007;14(5):517-528.

S5. Merege-Filho CAA, Otaduy MCG, de Sá-Pinto AL, et al. Does brain creatine content rely on exogenous creatine in healthy youth? A proof-of-principle study. *Applied Physiology, Nutrition, and Metabolism.* 2017;42(2):128-134.

S6. Pires L, Forbes S, Candow D, Machado M. Creatine supplementation on cognitive performance following Creatine supplementation on cognitive performance following exercise in female Muay Thai athletes exercise in female Muay Thai athletes. *J Soc NeuroSports*. 2020;1(6)

S7. Turner CE, Byblow WD, Gant N. Creatine supplementation enhances corticomotor excitability and cognitive performance during oxygen deprivation. *Journal of Neuroscience*. 2015;35(4):1773-1780.

S8. Rawson ES, Lieberman HR, Walsh TM, Zuber SM, Harhart JM, Matthews TC. Creatine supplementation does not improve cognitive function in young adults. *Physiology & behavior*. 2008;95(1-2):130-134.

S9. Borchio L, Machek SB, Machado M. Supplemental creatine monohydrate loading improves cognitive function in experienced mountain bikers. *The Journal of sports medicine and physical fitness*. 2020;60(8):1168-1170.

S10. Benton D, Donohoe R. The influence of creatine supplementation on the cognitive functioning of vegetarians and omnivores. *British journal of nutrition*. 2011;105(7):1100-1105.

trial.
